# IL-1β Promotes Stemness of Tumor Cells by Activating Smad/ID1 Signaling Pathway

**DOI:** 10.7150/ijms.44285

**Published:** 2020-05-18

**Authors:** Lin Lu, Peipei Wang, Yonghong Zou, Zhiqiang Zha, Haowei Huang, Mingmei Guan, Yong Wu, Guolong Liu

**Affiliations:** 1 Department of Medical Oncology, Guangzhou First People's Hospital, School of Medicine, South China University of Technology, Guangzhou, Guangdong, China, 510180; 2 Department of Medical Oncology, Guangzhou First People's Hospital, Guangzhou Medical University, Guangzhou, Guangdong, China, 510180; 3 Department of Gynecology and Obstetrics, Ji'an City Center People's Hospital, Jiangxi, China, 343000

**Keywords:** IL-1β, head and neck squamous cell carcinoma (HNSCC), melanoma, stemness, Smad/ID1 signal pathway

## Abstract

**Background**: IL-1β is reported to be involved in cancer development and distant metastasis. However, the underlying mechanism of IL-1β upon malignant behaviors remains largely unknown. In this study, we aimed to study whether IL-1β could enhance the stemness traits of tumor cells.

**Methods**: The concentrations of serum IL-1β in head and neck squamous cell carcinoma (HNSCC) and melanoma patients were detected using ELISA assay. The effect and mechanisms of IL-1β on tumor cell growth, migration, invasion and stemness characters were studied using HNSCC cell SCC7 and melanoma cell B16-F10. The underlying mechanisms were further explored.

**Results**: Enhanced concentrations of IL-1β were positively correlated with advanced tumor stage in both HNSCC and melanoma patients. IL-1β treatment led to a significant increase in tumor growth both in vitro and in vivo. IL-1β stimulation promoted cell proliferation, colony formation and tumorigenicity. In addition, IL-1β-stimulated tumor cells gained enhanced capabilities on wounding healing and invasion capabilities. Moreover, IL-1β stimulation promoted the stem-like capabilities of both HNSCC cells and melanoma cells, including the enrichment of aldehyde dehydrogenase^+^ (ALDH^+^) cells, up-regulation of stem cell related markers Nanog, OCT4, and SOX2, sphere formation and chemoresistance. Mechanistically, IL-1β treatment promoted the phosphorylation of Smad1/5/8 and activated its downstream target inhibitor of differentiation 1 (ID1). Silencing ID1 abrogated sphere formation and upregulated expression of stemness genes which were induced by IL-1β stimulation.

**Conclusion**: Our data demonstrates that IL-1β promotes the stemness of HNSCC and melanoma cells through activating Smad/ID1 signal pathway.

## Background

Local recurrence and distant metastasis are the major limitations for the failure of current cancer therapies. It was reported that cancer stem cells (CSCs) played a critical role in the treatment failure and were responsible for tumor relapse and metastasis 1, 2. CSCs are characterized by cellular heterogeneity, self-renewal, and multi-differential capabilities and resistant to conventional chemo and radio-therapy. In addition, CSCs are failed to express differentiated tumor antigens and hence insensitive to routine antitumor immunotherapies, which are mostly designed to target antigens on differentiated tumor cells 3-5. Thus, it is of great importance to explore factors that affect the functions of CSCs and design strategies specifically targeting CSCs.

An extensive crosstalk occurs between CSCs and tumor microenvironment 6-8. The tumor microenvironment is essential for the maintenance of stem cell-like capabilities of tumor cells 8. Soluble factors, such as cytokines, secreted by cells originated in the tumor microenvironment, stimulate self-renewal of CSCs and preserve the undifferentiated state of the cells 7, 9-11. Thus, it may generate new information for targeting CSC therapy to investigate cytokines that regulate CSCs in cancer microenvironment.

We previously reported that dendritic cells (DCs) pulsed with cancer stem cell lysates (CSC-DC) mediate specific humoral immunity against CSCs in murine squamous cell carcinoma and melanoma models 4, 12. We found that the level of IL-1β was significantly decreased in CSC-DC-treated mice, indicating that there might be a cross-talk between IL-1β and CSCs. IL-1β, belongs to IL-1 family, is mainly secreted by activated macrophages and monocytes. IL-1β participated in inflammatory processes, tumor invasiveness and metastasis 13-17. Studies have shown that IL-1β plays important roles in regulating the functions of stem cells. IL-1β stimulated the self-renewal of intestinal stem cells and induced the transition of these cells to CSCs 18. Wang et al found that combination of IL-1β and TGF-β induced the glioma neurosphere formation and promoted the malignant biological behaviors of glioma cells 19. However, the roles of IL-1β in the stemness maintenance of HNSCC and melanoma are largely unknown. In this study, we determined the impact of IL-1β on the stemness of squamous cell carcinoma and melanoma cells and explored the underlying mechanisms by which IL-1β maintains the stemness of CSCs.

## Methods

### Ethical statement and characteristics of patients

This study involved in human peripheral blood. Ethics Committee of Guangzhou First People's Hospital approved this study. All the patients included in this study were informed and consents were signed. There were 16 cases of healthy donors, 66 cases of HNSCC patients and 54 cases of melanoma patients enrolled in this study between January 2018 and August 2019.

### Mice and Ethical statement

Female C3H/HeNCr MTV (C3H) mice and C57BL/6 (B6) mice at the age of 6-8 weeks were purchased from Vital River Laboratory Animal Technology Co, Ltd (Beijing, China). Mice were housed in specific pathogen-free condition at the animal facility of South China University of Technology. All animal experiments conformed to our animal protocols approved by the Animal Care and Use Committee of South China University of Technology.

### ELISA

Peripheral blood was collected from cancer patients and healthy donors. After centrifugation, the serum samples were obtained and the concentration of IL-1β were assessed by ELISA assay according to the manufacturer's instructions (R&D Systems, USA). The ELISA assay was performed in triplicate.

### Cell Culture

Mouse squamous carcinoma cell line SCC7 and melanoma cell line B16-F10 were purchased from Cellcook Biotech Co.,Ltd (Guangzhou, China). Cells were cultured in RMPI 1640 supplemented with 10% heat-inactivated fetal bovine serum, 100 μg/mL streptomycin, 100 U/mL penicillin. For the sphere formation assays, tumor cells were cultured in serum free medium consisting of DMEM supplemented with 20 ng/ml EGF (Peprotech, USA), 20 ng/ml FGF (Perotech, USA), 2 mL of 2% B27 supplement (Gibco, USA), 4 μg/ml insulin, 100 μg/mL streptomycin and 100 U/mL penicillin. For some experiments, tumor cells were treated with 10 ng/ml IL-1β or 100 ng/ml IL-1 receptor antagonist (IL-1 Ra). For the ID1 siRNA transfection assay, tumor cells were seeded and allowed to grow in culture medium for 24h. Then tumor cells were treated with ID1 siRNA (siID1) or siRNA negative control (siNC, GenePharma, Shanghai) using Lipofectamine RNAiMAX (Thermo Fisher).

### MTT assay

Cell proliferation capabilities were measured using MTT assay. Briefly, tumor cells were seeded in a 96-well plate at a density of 5 x 10^4^ cells/well. After being incubated with or without IL-1β for 72 hours, the tumor cells were treated with 50 μL of MTT solution (1 mg/ml). The resulting crystals were dissolved in DMSO and measured at a wavelength of 570 nm with a multi-mode microplate reader (PerkinElmer, USA). The experiment was repeated in triplicate.

### Colony formation assay

Cells were placed in 6-well plates at a concentration of 500 cells per well. Cell culture medium supplemented with or without IL-1β was changed every 3 days. After incubation for 14 days, the cells were fixed using 75% ethanol, followed by staining with 0.5% crystal violet. After air dried at room temperature, the cells were visualized under a microscope. Colonies comprising 50 or more cells were counted. Each experiment was repeated at least 3 times.

### Tumorigenicity

The squamous cell carcinoma SCC7 is syngeneic to C3H mice. The melanoma cell line B16-F10 is syngeneic to B6 mice. The mice were randomly divided into each group with at least 5 mice/group. Tumor cells stimulated with or without IL-1β were inoculated into the right flank of the syngeneic mice at the number of 5 x 10^4^. The tumor size was measured every 3 days and the tumor volume was calculated using formula: (LxW^2^)/2. The experiment was repeated in triplicate.

### Wound healing assay

Cells stimulated with or without IL-1β were placed into 6-well plate and cultured for the formation of monolayer. Scratched wounds were then prepared by scraping the cell layer on each culture plate using a tip of 10 μL pipette, and the debris was removed by washing the cells with PBS. Wounded cultures were incubated for 24 hours in serum-free medium. Then 3 fields of view were randomly picked from each of the scratched wound and observed by microscopy to evaluate cell migration ability.

### Cell invasion

To perform the matrigel invasion assay, 24-well plates were filled with 500 μL of complete medium with 20% FBS. Tumor cells stimulated with or without IL-1β (5 x 10^4^) in 100 μL medium were placed into polycarbonate membrane insert (8-μm, Corning, USA)) coated with a thin layer of 0.5 mg/mL Matrigel Basement Membrane Matrix (BD Biosciences, Bedford, MA). After being incubated for 48 hours, the insert membranes were fixed with 75% methanol. After removing the cells on the upper surface, the cells invaded into the lower surface were stained with 0.5% crystal violet supplemented with 20% methanol. The results were calculated by counting the stained cells under an inverted microscope (10 fields per membrane). Each experiment was repeated at least 3 times.

### Chemoresistance

Indicated SCC7 cells and B16-F10 cells (5,000 cells) were seeded in a 96-well plate. SCC7 cells were treated with 10 μg/ml Paclitaxel (PTX), while B16-F10 cells cells were treated with 0.5 μg/ml Doxorubicin (DOX). The cell viabilities were assessed using MTT assay 24h after the treatment with PTX or DOX. The OD value was measured at a wavelength of 570 nm with a multi-mode microplate reader (PerkinElmer, USA).

### ALDEFLUOR Assay

The fluorogenic dye-based ALDEFLUOR assay kit (StemCell Technologies) was used to detect the activity of ALDH as previously described^4^, ^12^. Briefly, after being stimulated with or without IL-1β, tumor cells were resuspended at a concentration of 1 x 10^6^ cells/ml and incubated with ALDEFLUOR substrate. Cells were then analyzed using FACSAria III flow cytometer (BD, USA). Tumor cells treated with ALDH inhibitor diethylaminobenzaldehyde (DEAB) were used as negative control. The experiment was repeated in triplicate.

### Real time quantitative PCR

Total RNA was extracted with TRIzol solution (Invitrogen, USA) as previously described 20, 21. To synthesize cDNA, the total RNA was treated with 0.5 μg of Oligo(dt), 200 U M-MLV reverse transcriptase, 25 U RNase inhibitor and 2.5 mM dNTP using a 25 μl reaction volume. The resulting cDNA was used to measure the relative expression of stem cell genes and internal control GAPDH using SYBR Green master mix (Invitrogen) on a 7500 FAST Real-time PCR system (Applied Biosystems). The primers used are as follows, 5'-3'(F: GCACGCCAGACTTACCTGTC) and 5'-3'(R: CCTCCTCAGTTGCAGGATTAAAG) for ALDH; 5'-3'(F: CAAAGGATGAAGTGCAAGCG) and 5'-3'(R: CCAGATGCGTTCACCAGATAG ) for Nanog; 5'-3'(F: CAGAGAAAACCTGAGGGCGG) and 5'-3'(R: GACTTTTGCGAACTCCCTGC) for SOX2; 5'-3'(F: TAGGTGAGCCGTCTTTCCAC) and 5'-3'(R: GCTTAGCCAGGTTCGAGGAC) for OCT4. Each experiment was repeated at least 3 times using cDNA samples from separate reverse transcription reactions.

### Western blotting

The western blotting was performed using routine protocol as previously described 21. Antibodies against Nanog, SOX2, OCT4, Smad 1, p-Smad 1/5/8 and GAPDH were purchased from Cell Signaling Technologies and used at 1:1000 dilution. ID1 antibody was purchased from Proteintech and used at 1:1000 dilution.

### Sphere formation

Tumor cells were plated in 6-well ultra-low attachment plates in serum free culture medium with or without IL-1β stimulation. The sphere formation was monitored from day 7. Then the sphere was counted on day 14 under a Leicia inverted microscope.

### Statistical analysis

Quantitative values were expressed as mean ± SD. Unpaired Student's t-test (2 cohorts) or one-way analysis (> 2 cohorts) were used to value the differences between experiment groups. A two-tailed P value < 0.05 was considered statistically significant. All the statistical analyses were performed using Graphpad Prism 7 (GraphPad Software, Inc., USA).

## Results

### Serum IL-1β concentration is correlated with advanced stages of HNSCC patients and melanoma patients

**Figure 1:**
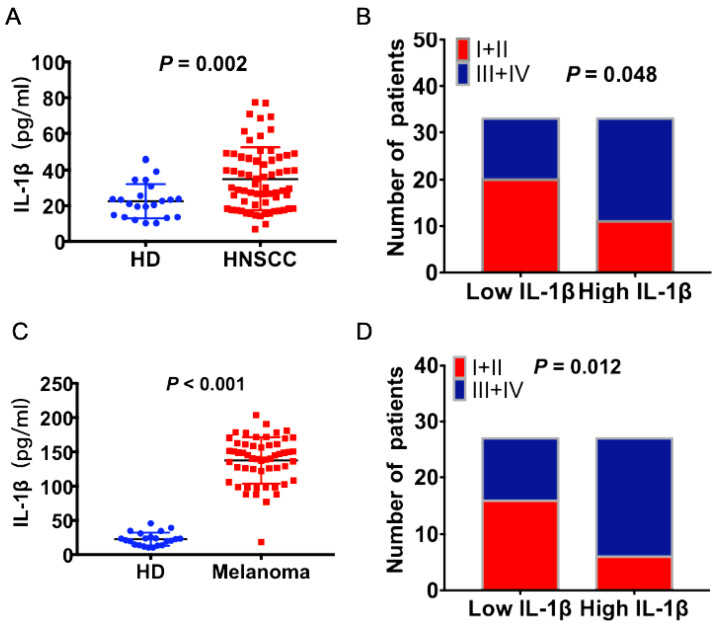
** Increased expression levels of IL-1β were correlated with advanced tumor stage in HNSCC and melanoma patients.** IL-1β concentrations were detected using ELISA assay. (A) The concentrations of IL-1β in HNSCC patients and healthy donors (HD) were compared. (B) The correlation between IL-1β and tumor stage of HNSCC patients was assessed. (C) The concentrations of IL-1β in melanoma patients and healthy donors were compared. (B) The correlation between IL-1β and tumor stage of melanoma patients was assessed.

The concentrations of IL-1β in the peripheral blood of HNSCC patients and health donors (HD) were detected using ELISA assay. As shown in Figure 1A, the concentrations of IL-1β were much higher in the HNSCC patients than healthy donors (P = 0.002, Figure 1A). The patients were divided into two groups based on the median value of IL-1β concentration: Low IL-1β and high IL-1β. Enhanced IL-1β was positively correlated with advanced tumor stage (P = 0.048, Figure 1B). Similarly, the concentration of IL-1β was much higher in melanoma patients than healthy donors (P < 0.001, Figure 1C). And there was positive correlation between IL-1β concentration and tumor stages (P = 0.012, Figure 1D). Most of the patients with low IL-1β concentrations were staged as TNM I+II, while most of the patients with high IL-1β concentrations were staged as TNM III+IV.

### IL-1β stimulation promotes tumor cell growth in vitro and in vivo

**Figure 2:**
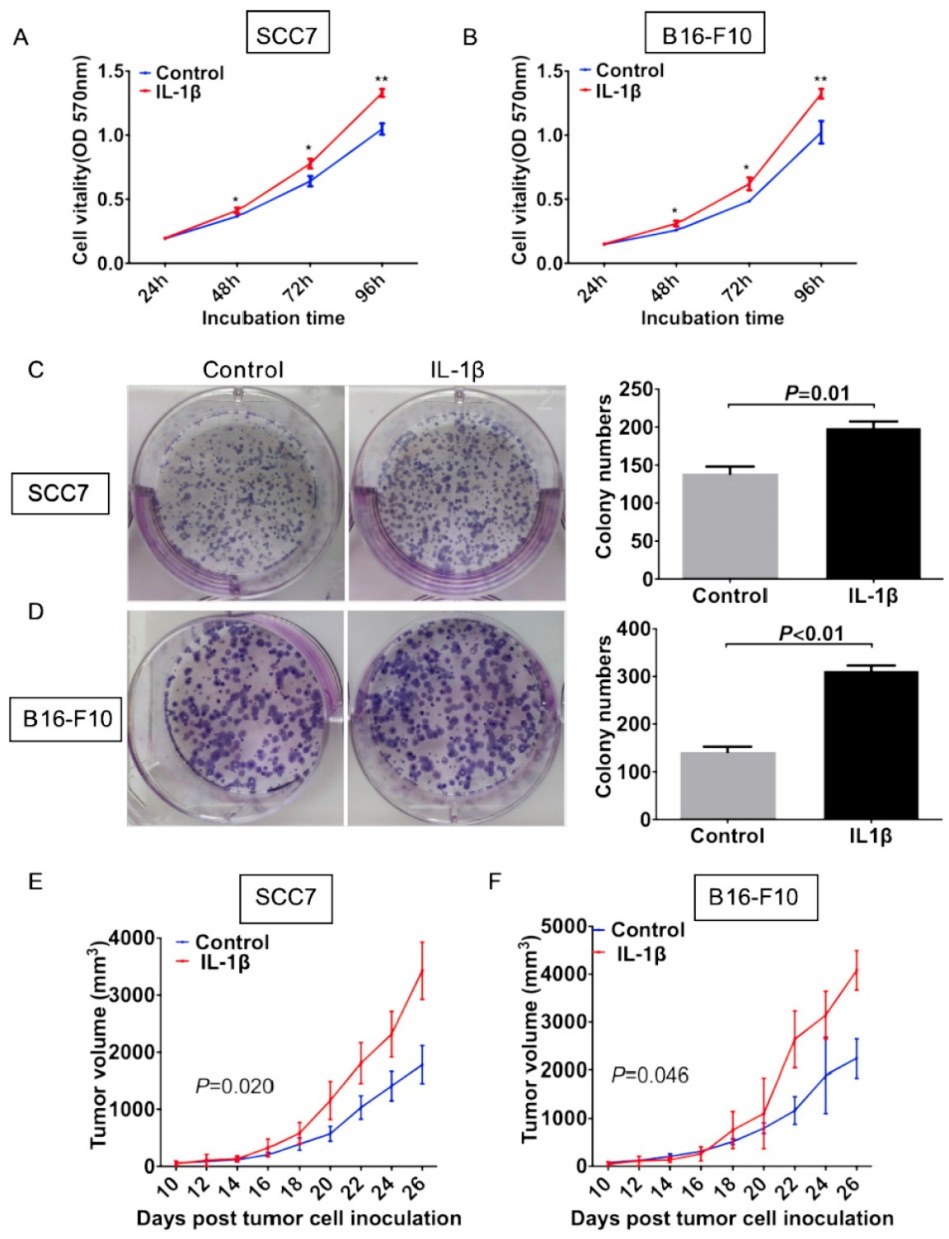
** IL-1β stimulation promotes tumor growth in vitro and in vivo.** SCC7 cells and B16-F10 cells were treated with 100 ng/ml IL-1β. Cell proliferation was evaluated using MTT assay. IL-1β stimulation enhanced viabilities of SCC7 cells (A) and B16-F10 cells (B). IL-1β significantly enhanced the colony formation capability of SCC7 cell C) and B16-F10 cells (D). Representative images of colony formation after IL-1β stimulation were demonstrated on the left. IL-1β stimulation could increase the tumorigenicity of SCC7 cells (E) and B16-F10 cells (F) in vivo. (*, P < 0.05; **, P < 0.01; ***, P < 0.001)

To evaluate the effect of IL-1β on the cell viability, we carried out both cell proliferation assay and colony formation assay. In the cell proliferation assay, the growth rate of control SCC7 cells was significantly lower than that of the IL-1β-stimulated SCC7 cells starting from 48 hours after incubation (P < 0.05, Figure 2A). Similar experiment in the melanoma cells also showed that cells under IL-1β stimulation possessed more stronger proliferation ability (P < 0.05, Figure 2B). As demonstrated in Figure 2C, IL-1β-stimulated SCC7 cells showed a significant enhancement in colony formation as compared with control SCC7 cells (P = 0.01, Figure 2C). Similar results were also obtained from the B16-F10 melanoma cells (P < 0.01, Figure 2D).

We next investigated the effect of IL-1β on tumor growth in vivo. Squamous cell carcinoma SCC7 stimulated with or without IL-1β were inoculated subcutaneously. As shown in Figure 2E, volumes of tumors generated from IL-1β-stimulated cells were much larger than those generated from untreated tumor cells (Figure 2E, P = 0.020). Similar results were generated from the melanoma model. With the stimulation of IL-1β, B16-F10 tumor cells formed larger size of tumors than untreated tumor cells (Figure 2F, P = 0.046). These data suggest that IL-1β stimulation could promote tumor cell growth both in vitro and in vivo.

### IL-1β stimulation promotes tumor cell migration and invasion

**Figure 3:**
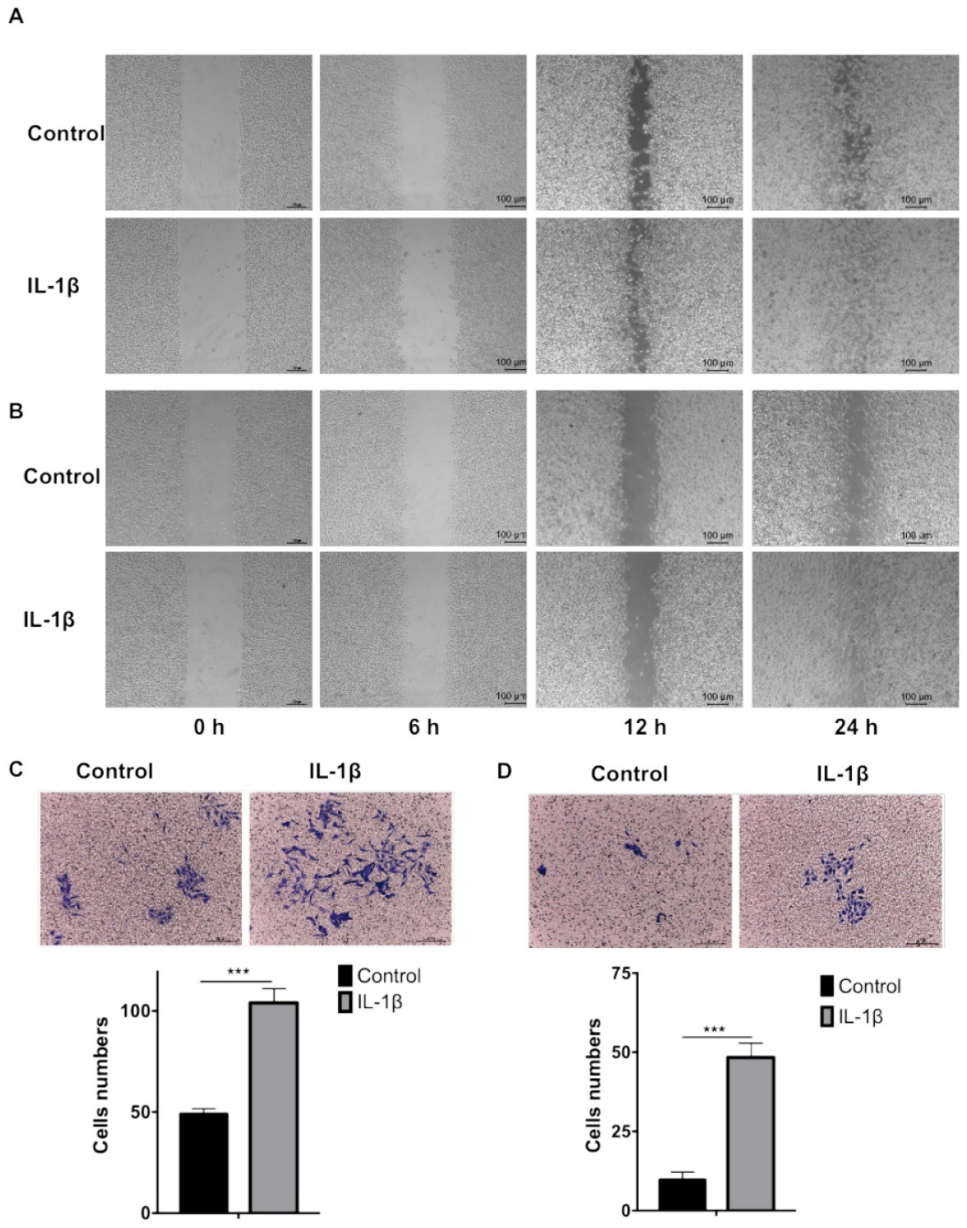
** IL-1β stimulation enhances the migration and invasion of HNSCC and melanoma cells.** The wound healing assay demonstrated that IL-1β stimulation enhanced the migration capabilities of SCC7 cells (A) and B16-F10 cells (B). Transwell assay demonstrated that the invasion capabilities of SCC7 cells (C) and B16-F10 cells (D) were enhanced after IL-1β stimulation. ( ***, P < 0.001)

We firstly carried out wound healing assay to evaluate the effect of IL-1β on cell migration. As shown in Figure 3A and B, the cell migration started from 6 h and the devoid edges coalesced 24 h after scraping under the stimulation of IL-1β. The IL-1β-stimulated SCC7 (Figure 3A) and B16-F10 (Figure 3B) cells demonstrated stronger migration capability than the control tumor cells did.

Transwell assay was employed to investigate the effect of IL-1β on tumor cells invasion. As shown in Figure 3C and D, IL-1β-stimulated SCC7 cells and B16-F10 cells invade into the lower compartments much more frequently than the control SCC7 cells and B16-F10 cells.

### IL-1β stimulation enhances the stemness of HNSCC cells and melanoma cells

**Figure 4:**
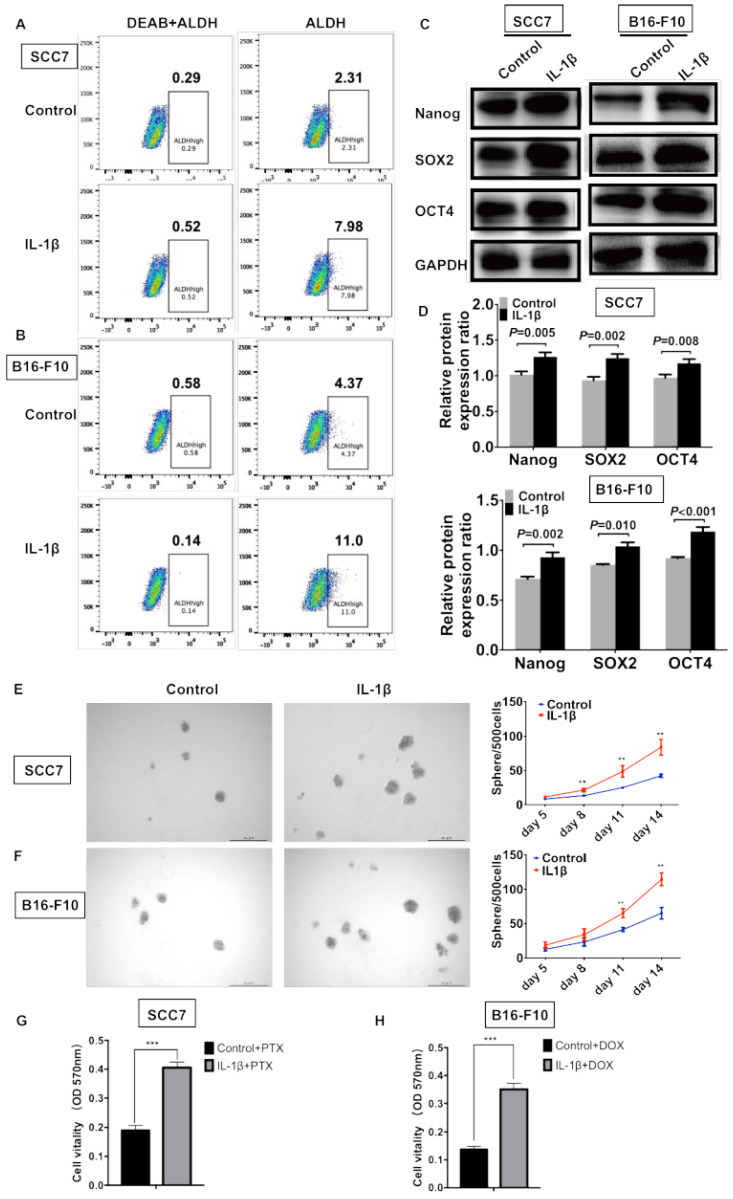
** IL-1β stimulation promotes the stemness phenotype of squamous cell carcinoma cells and melanoma cells.** IL-1β stimulation increased the percentage of ALDH^high^ CSCs. The results analyzed by flow cytometry in squamous cell carcinoma cells (A) and melanoma cells (B). IL-1β stimulation upregulated the expression of stem cell genes Nanog, SOX2 and OCT4 in squamous cell carcinoma and melanoma models (C, D). (D) The relative protein expression ratio of variable stemness genes were normalized to internal control. IL-1β enhance the sphere formation capabilities of SCC7 cells (E) and B16-F10 cells (F). Representative images of sphere formation were shown on the left. Chemoresistance was analyzed using MTT assay after IL-1β stimulation. (G) IL-1β-stimulated SCC7 cells were more resistant to paclitaxel (PTX) than control cells. (H) IL-1β-stimulated B16-F10 cells were more resistant to doxorubicin (DOX).

ALDEFLUOR/ALDH has been widely used as a single marker to identify and isolate CSCs from various tumors, including squamous cell carcinoma and melanoma 3, 5. To determine whether IL-1β could enhance the enrichment of CSCs, flow cytometry was performed to assess the percentage of ALDH^high^ cells. As shown in Figure 4A, 2.31% of the control SCC7 cells were the ALDH^high^ subpopulation. Approximately 7.98% of the IL-1β-stimulated SCC7 tumor cells were ALDH^high^. In the B16-F10 melanoma model, the IL-1β stimulation significantly increased the percentage of ALDH^high^ cells (11% vs. 4.37%, Figure 4B).

Western blotting assay was carried out to assess the levels of stem cell genes Nanog, SOX2, and OCT4. In the squamous cell carcinoma, the expression of Nanog (P = 0.005), SOX2 (P = 0.002), and OCT4 (P = 0.008) was significantly increased after IL-1β stimulation (Figure 4C, D). In the melanoma model, the IL-1β-stimulated B16-F10 cells expressed higher levels of Nanog (P = 0.002), SOX2 (P = 0.010), and OCT4 (P < 0.001) as compared with non-stimulated cells (Figure 4C, D).

Sphere formation represents the self-renewal capability of CSCs. SCC7 and B16-F10 cells were cultured using serum-free medium supplemented with or without IL-1β. Compared to control tumor cells, the IL-1β-stimulated tumor cells possessed enhanced sphere formation ability (Figure 4E and F). IL-1β-stimulated tumor cells formed much more spheres than those in control cells in both squamous cell carcinoma (P < 0.01, Figure 4E) and melanoma cells (P < 0.01, Figure 4F).

Cancer cell stemness is often associated with drug resistance, thus we further studied whether IL-1β stimulation could increase the chemoresistance of SCC7 cells and B16-F10 cells. As shown in Figure 4G and 4H, after IL-1β stimulation, SCC7 cells and B16-F10 cells were more resistant to paclitaxel (PTX) and doxorubicin (DOX), respectively (P < 0.001). Together, our results demonstrate that IL-1β treatment promotes the stem cell-like capabilities of both HNSCC and melanoma cells.

### IL-1β stimulation activates Smad 1/5/8 and ID1 to maintain the stenmess of tumor cells

**Figure 5:**
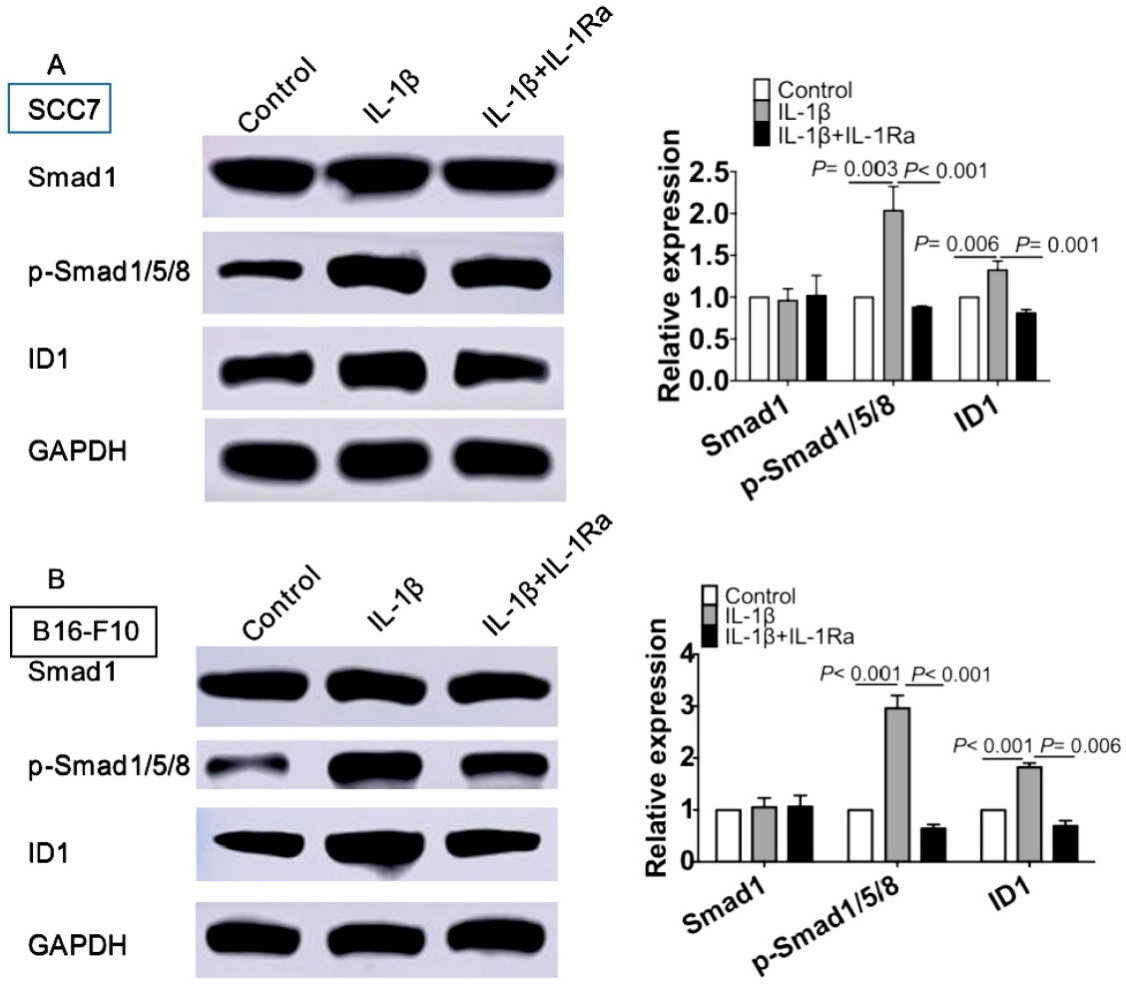
** IL-1β stimulation activates Smad1/5/8 and ID1 signaling pathway.** SCC7 cells and B16-F10 cells were treated with IL-1β (10 ng/ml) or IL-1 Ra (100 ng/ml). The protein levels of Smad1, phosphorylated-Smad1/5/8 and ID1 were analyzing using western blot in SCC7 cells (A) and B16-F10 cells (B). IL-1β stimulation significantly upregulated the expression of p- Smad1/5/8 and ID1 in SCC7 cells and B16-F10 cells.

We further investigated the mechanisms underlying IL-1β treatment on enhancing stemness of squamous cell carcinoma and melanoma. IL-1β stimulation escalated the phosphorylation of Smad 1/5/8 and up-regulated its downstream target ID1 (P < 0.05, Figure 5A). A similar result was obtained in IL-1β-treated B16 melanoma cells (P < 0.05, Figure 5B). Together, our data support the notion that IL-1β stimulation promoted the activation of Smad/ID1 signaling pathway.

**Figure 6:**
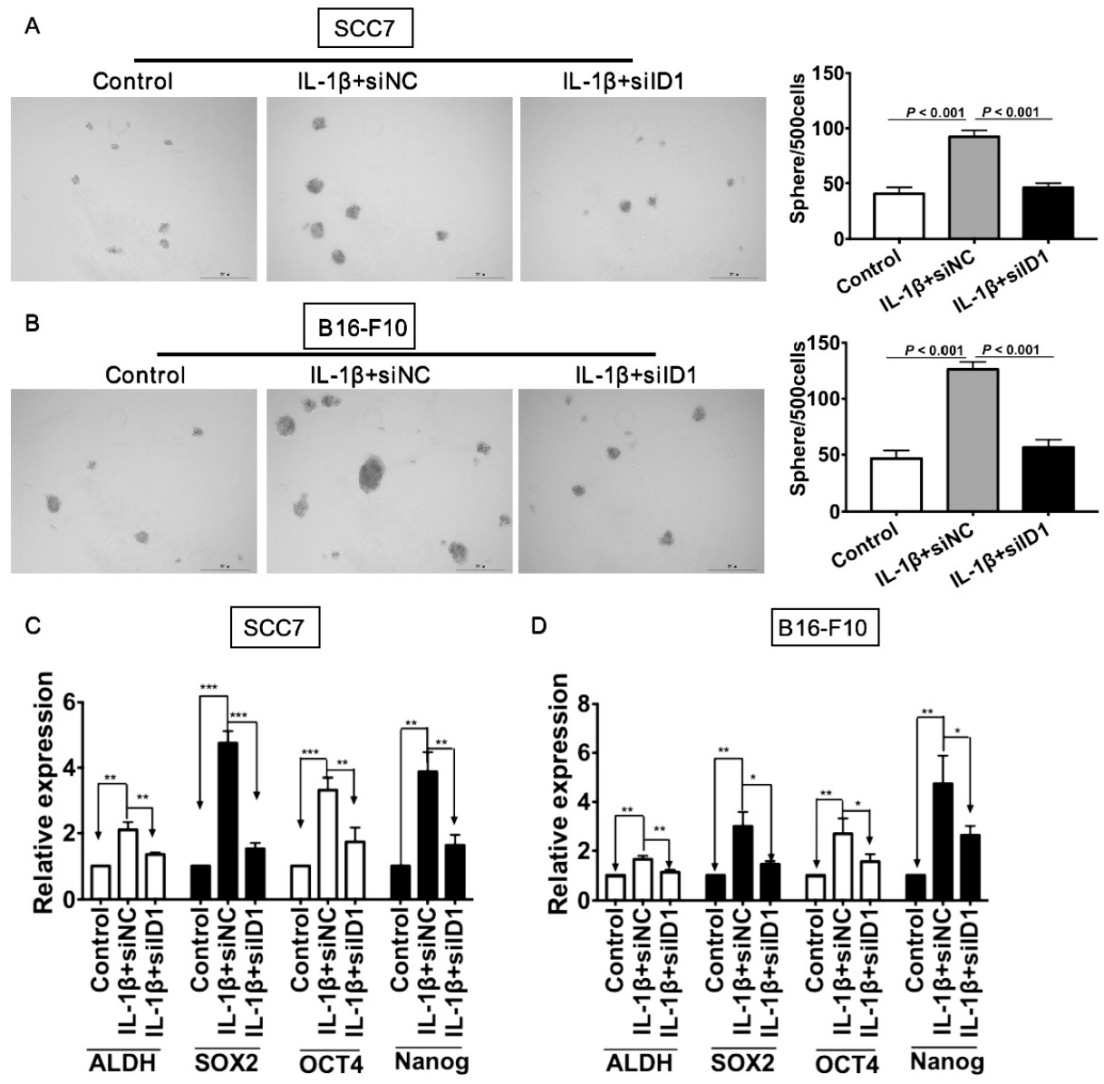
** Silencing ID1 reverses IL-1β stimulation-induced tumor cell stemness.** ID1 was knocked down with siID1, while siNC was used as a negative control. (A and B) Silencing ID1 inhibits sphere formation capability induced by IL-1β stimulation in SCC7 cells (A) and B16-F10 cells (B). Representative images of sphere formation were displayed on the left. The sphere numbers were calculated and shown on the right**.** Silencing ID1 reverses IL-1β stimulation-induced upregulation of stemness genes, including ALDH, SOX2, OCT4 and Nanog in SCC7 cells (C) and B16-F10 cells (D).

We next investigated whether ID1 contributed to IL-1β treatment-enhancing stemness. Silencing of ID1 significantly abrogated the tumor sphere formation induced by IL-1β stimulation (P < 0.05, vs. all other groups, Figure 6A). Experiments were also conducted in melanoma cells. As shown in Figure 6B, the sphere formation capability of tumor cells subjected to IL-1β stimulation and ID1 silence was significantly reduced (P < 0.05, vs. all other groups). Furthermore, ID1 knockdown reversed up-regulation of stem cell related markers induced by IL-1β stimulation in SCC7 cells (P < 0.05, Figure 6C) and B16-F10 tumor cells (P < 0.05, Figure 6D).

## Discussion

CSCs are generally accepted as the source of tumor relapse and distant metastasis. Cells in this population gain self-renewal and more aggressive malignant behaviors, as well as more resistance to current anti-tumor therapies 5, 22, 23. Currently, strategies specifically targeting CSCs remain unsatisfactory. Our previous studies demonstrated that CSC-DC (dendritic cells pulsed with cancer stem cell lysates) vaccine mediates anti-CSC immunity by directly targeting CSCs and inhibits tumor growth and distant metastasis 4, 12. These findings were verified in both melanoma and squamous cell carcinoma models.

It is generally accepted that tumor regression and progression are determined by both intrinsic and extrinsic components of cells. The latter are mainly determined by tumor microenvironment 24. Cytokines play an important and complex biological role in regulating the number and functions of CSCs in tumor microenvironment 7, 25. Thus, manipulating the levels of cytokines that regulate CSCs might enable the treatment efficacy of current immune strategies.

IL-1β, as one of the most potent pro-inflammatory cytokines, is generated by tumor cells and immune cells, such as macrophages, in tumor microenvironment 26. IL-1β is increased in a variety of tumors and its expression is associated with the tumor development and progression 13, 15-17. IL-1β could enhance the tumor invasion and distant metastasis 13, 27, 28. Accordingly, our data revealed that enhanced concentrations of IL-1β in HNSCC and melanoma patients were positively correlated with advanced tumor stage. However, there are few reports investigating the effect of IL-1β on stem cells and CSC functions. Application of soluble IL-1 receptors and IL-1 receptor antagonist or IL-1β neutralizing antibodies inhibit acute myelogenous leukemia (AML) blast colony formation, which is reversed by IL-1β 29. In the presence of IL-1β, normal intestinal epithelial cells could form spheres and large colonies, as well as expressing more stem cell genes Bmi-1, Lgr-5, Nanog and β-catenin 18. Li et al found that IL-1β could promote tumor growth and invasion through the activation of CSCs and epithelial-mesenchymal transition (EMT) 30.

In the current study, we demonstrated that IL-1β could promote the colony formation, cell proliferation and tumorigeneicity, which indicating that IL-1β stimulating induced tumor growth both in vitro and in vivo. In addition, IL-1β-stimulated tumor cells displayed enhanced migratory and invasive capabilities. IL-1β stimulation could enrich the percentage of ALDH^high^ population in both SCC7 cells and B16-F10 cells. The increased expression of ALDH after IL-1β stimulation might confer more tumor cells with stem cell capability. In addition, we found that IL-1β stimulation promoted the sphere formation ability, up-regulated expression of stem cell genes (SOX2, OCT4 and Nanog) and increased chemoresistance, suggesting that IL-1β could promote the stemness of CSCs. Consistently, Li et al previously reported that IL-1β can enhance the sphere formation concomitant with up-regulation of stemness markers Bmi1 and nestin in colon cancers 30. Taken together, these data demonstrate that IL-1β stimulation promotes the stemness capabilities of squamous carcinoma cells and melanoma cells.

Inhibitors of differentiation 1(ID1) is reported to be upregulated in malignant tumor cells 31, 32. Increased ID1 expression was correlated with advanced stage and poor overall survival in esophageal squamous cell carcinoma 31. ID1 maintained the neural and embryonic stem cells properties 33, 34. Silencing of ID1 impaired CSC-like capability and inhibited EMT traits in colorectal cancer 32. ID1 is a downstream transcription factor for Smad1/5/8/, and activation of Smad1/5/8 signaling pathway transcriptionally upregulated ID1 expression to promote migration and invasion of esophageal squamous cancer cells 35. The phosphorylation of Smad1/5/8/ and activation of ID1 contributed to angiogenesis and tumor growth in lung cancer^36^. Consistently, our study demonstrated that phosphorylation of Smad1/5/8 and activation of ID1 was upregulated with IL-1β stimulation. Moreover, knockdown of ID1 could abrogate IL-1β stimulation-prompted stem cell-like properties of HNSCC and melanoma cells. Here, we revealed the underlying mechanism of IL-1β-promoted tumor cell stemness by studying BMP1/5/8 and ID1 signaling pathway, but not TGF-β/Smad2/3 signaling pathway. The effects of IL-1β on TGF-β/Smad signal pathway are controversial. There was interaction between IL-1β and TGF-β in human mesenchymal stem cell. IL-1β repressed TGF-β signal 37. Ohta et al revealed that IL-1β suppressed the activation of TGF-β/Smad2/3 to downregulated the expression of nerve growth factor 38. However, Liu et al found that IL-1β could enhance TGF-β1-induced epithelial mesenchymal transition in lung adenocarcinoma cell 39. Luo et al found that IL-1β modulated the TGF-β signal dynamically in the proximal tubular cell transforming 40. In this study, our data indicate the IL-1β stimulation could enhance the stemness of HNSCC and melanoma cells through activating Smad1/5/8 and ID1 signaling pathway. Our next work would further determine whether TGF-β/Smad2/3 signaling pathway play more important role than BMP1/5/8/ ID1 signaling pathway.

Remodeling tumor environment could help eliminate CSCs, which might be an effectively immunotherapy and promote the anti-tumor immunity of CSC-DC 22, 41-43. Ginestier et al demonstrated that blockade of IL-8 receptor CXCR1 could help target breast CSCs^41^. Kim et al found that anti-IL-6 inhibits CSCs in breast tumor cells^42^. Korkaya et al suggested that blocking cytokines and their corresponding receptors inhibits the self-renewal of CSCs and respective trials have been initiated to block CSCs 43. Our study demonstrated that IL-1β could enhance the stemness of tumor cells, indicating that blockade of IL-1β might be an effective strategy to enhance the anti-tumor immunity of CSC-DC. Thus, in our future study, we will determine the therapeutical efficacy of IL-1β neutralization in HNSCC and melanoma.

## Conclusion

In conclusion, our study demonstrated that IL-1β could enhance the stemness of squamous cell carcinoma and melanoma via activating Smad/ID1 pathway. These findings provide evidence that regulating the expression of IL-1β in tumor microenvironment might be an effective way to specifically targeting CSCs and could be used as an adjuvant for tumor immunotherapies.

## Abbreviations

CSCs: cancer stem cells
HNSCC: head and neck squamous cell carcinoma
ELISA: Enzyme linked immunosorbent assay
HD: healthy donors
ID1: inhibitor of DNA binding 1
CSC-DC: cancer stem cell lysates pulsed dendritic cells vaccine
ALDH: aldehyde dehydrogenase

## References

[1] Nandi S, Ulasov IV, Tyler MA, Sugihara AQ, Molinero L, Han Y (2008). Low-dose radiation enhances survivin-mediated virotherapy against malignant glioma stem cells. Cancer research.

[2] Dallas NA, Xia L, Fan F, Gray MJ, Gaur P, van Buren G 2nd (2009). Chemoresistant colorectal cancer cells, the cancer stem cell phenotype, and increased sensitivity to insulin-like growth factor-I receptor inhibition. Cancer research.

[3] Li Q, Lu L, Tao H, Xue C, Teitz-Tennenbaum S, Owen JH Generation of a novel dendritic-cell vaccine using melanoma and squamous cancer stem cells. Journal of visualized experiments: JoVE. 2014: e50561.

[4] Lu L, Tao H, Chang AE, Hu Y, Shu G, Chen Q (2015). Cancer stem cell vaccine inhibits metastases of primary tumors and induces humoral immune responses against cancer stem cells. Oncoimmunology.

[5] Ning N, Pan Q, Zheng F, Teitz-Tennenbaum S, Egenti M, Yet J (2012). Cancer stem cell vaccination confers significant antitumor immunity. Cancer research.

[6] Nassar D, Blanpain C (2016). Cancer Stem Cells: Basic Concepts and Therapeutic Implications. Annual review of pathology.

[7] Todaro M, Alea MP, Di Stefano AB, Cammareri P, Vermeulen L, Iovino F (2007). Colon cancer stem cells dictate tumor growth and resist cell death by production of interleukin-4. Cell stem cell.

[8] Scadden DT (2006). The stem-cell niche as an entity of action. Nature.

[9] Folkins C, Man S, Xu P, Shaked Y, Hicklin DJ, Kerbel RS (2007). Anticancer therapies combining antiangiogenic and tumor cell cytotoxic effects reduce the tumor stem-like cell fraction in glioma xenograft tumors. Cancer research.

[10] Francipane MG, Alea MP, Lombardo Y, Todaro M, Medema JP, Stassi G (2008). Crucial role of interleukin-4 in the survival of colon cancer stem cells. Cancer research.

[11] Liu S, Ginestier C, Ou SJ, Clouthier SG, Patel SH, Monville F (2011). Breast cancer stem cells are regulated by mesenchymal stem cells through cytokine networks. Cancer research.

[12] Hu Y, Lu L, Xia Y, Chen X, Chang AE, Hollingsworth RE (2016). Therapeutic Efficacy of Cancer Stem Cell Vaccines in the Adjuvant Setting. Cancer research.

[13] Apte RN, Dotan S, Elkabets M, White MR, Reich E, Carmi Y (2006). The involvement of IL-1 in tumorigenesis, tumor invasiveness, metastasis and tumor-host interactions. Cancer metastasis reviews.

[14] Germano G, Allavena P, Mantovani A (2008). Cytokines as a key component of cancer-related inflammation. Cytokine.

[15] Lewis AM, Varghese S, Xu H, Alexander HR (2006). Interleukin-1 and cancer progression: the emerging role of interleukin-1 receptor antagonist as a novel therapeutic agent in cancer treatment. Journal of translational medicine.

[16] Okamoto M, Liu W, Luo Y, Tanaka A, Cai X, Norris DA (2010). Constitutively active inflammasome in human melanoma cells mediating autoinflammation via caspase-1 processing and secretion of interleukin-1beta. The Journal of biological chemistry.

[17] Voronov E, Shouval DS, Krelin Y, Cagnano E, Benharroch D, Iwakura Y (2003). IL-1 is required for tumor invasiveness and angiogenesis. Proceedings of the National Academy of Sciences of the United States of America.

[18] Wang L, Liu Z, Li Y, Pappan L, Galliher-Beckley A, Shi J (2012). Pro-inflammatory cytokine interleukin-1beta promotes the development of intestinal stem cells. Inflammation research: official journal of the European Histamine Research Society [et al].

[19] Wang L, Liu Z, Balivada S, Shrestha T, Bossmann S, Pyle M (2012). Interleukin-1beta and transforming growth factor-beta cooperate to induce neurosphere formation and increase tumorigenicity of adherent LN-229 glioma cells. Stem cell research & therapy.

[20] Lu L, Pan K, Zheng HX, Li JJ, Qiu HJ, Zhao JJ (2013). IL-17A promotes immune cell recruitment in human esophageal cancers and the infiltrating dendritic cells represent a positive prognostic marker for patient survival. Journal of immunotherapy (Hagerstown, Md: 1997).

[21] Wang W, Lv L, Pan K, Zhang Y, Zhao JJ, Chen JG (2011). Reduced expression of transcription factor AP-2alpha is associated with gastric adenocarcinoma prognosis. PloS one.

[22] Pan Q, Li Q, Liu S, Ning N, Zhang X, Xu Y (2015). Concise Review: Targeting Cancer Stem Cells Using Immunologic Approaches. Stem cells (Dayton, Ohio).

[23] Saygin C, Matei D, Majeti R, Reizes O, Lathia JD (2019). Targeting Cancer Stemness in the Clinic: From Hype to Hope. Cell stem cell.

[24] Hui L, Chen Y (2015). Tumor microenvironment: Sanctuary of the devil. Cancer letters.

[25] Charafe-Jauffret E, Ginestier C, Iovino F, Wicinski J, Cervera N, Finetti P (2009). Breast cancer cell lines contain functional cancer stem cells with metastatic capacity and a distinct molecular signature. Cancer research.

[26] Dinarello CA (1996). Biologic basis for interleukin-1 in disease. Blood.

[27] Dinarello CA (2010). Why not treat human cancer with interleukin-1 blockade?. Cancer metastasis reviews.

[28] Song X, Voronov E, Dvorkin T, Fima E, Cagnano E, Benharroch D (2003). Differential effects of IL-1 alpha and IL-1 beta on tumorigenicity patterns and invasiveness. Journal of immunology (Baltimore, Md: 1950).

[29] Estrov Z, Kurzrock R, Estey E, Wetzler M, Ferrajoli A, Harris D (1992). Inhibition of acute myelogenous leukemia blast proliferation by interleukin-1 (IL-1) receptor antagonist and soluble IL-1 receptors. Blood.

[30] Li Y, Wang L, Pappan L, Galliher-Beckley A, Shi J (2012). IL-1beta promotes stemness and invasiveness of colon cancer cells through Zeb1 activation. Molecular cancer.

[31] Yuen HF, Chan YP, Chan KK, Chu YY, Wong ML, Law SY (2007). Id-1 and Id-2 are markers for metastasis and prognosis in oesophageal squamous cell carcinoma. British journal of cancer.

[32] Sun Y, Lai X, Yu Y, Li J, Cao L, Lin W (2019). Inhibitor of DNA binding 1 (Id1) mediates stemness of colorectal cancer cells through the Id1-c-Myc-PLAC8 axis via the Wnt/beta-catenin and Shh signaling pathways. Cancer management and research.

[33] Jung S, Park RH, Kim S, Jeon YJ, Ham DS, Jung MY (2010). Id proteins facilitate self-renewal and proliferation of neural stem cells. Stem cells and development.

[34] Romero-Lanman EE, Pavlovic S, Amlani B, Chin Y, Benezra R (2012). Id1 maintains embryonic stem cell self-renewal by up-regulation of Nanog and repression of Brachyury expression. Stem cells and development.

[35] Hu M, Cui F, Liu F, Wang J, Wei X, Li Y (2017). BMP signaling pathways affect differently migration and invasion of esophageal squamous cancer cells. International journal of oncology.

[36] Jia Y, Wang Z, Zang A, Jiao S, Chen S, Fu Y (2016). Tetramethylpyrazine inhibits tumor growth of lung cancer through disrupting angiogenesis via BMP/Smad/Id-1 signaling. International journal of oncology.

[37] van den Akker GG, van Beuningen HM, Vitters EL, Koenders MI, van de Loo FA, van Lent PL (2017). Interleukin 1 β-induced SMAD2/3 linker modifications are TAK1 dependent and delay TGFβ signaling in primary human mesenchymal stem cells. Cellular signalling.

[38] Ohta M, Chosa N, Kyakumoto S, Yokota S, Okubo N, Nemoto A (2018). IL-1β and TNF-α suppress TGF-β-promoted NGF expression in periodontal ligament-derived fibroblasts through inactivation of TGF-β-induced Smad2/3- and p38 MAPK-mediated signals. International journal of molecular medicine.

[39] Liu X (2008). Inflammatory cytokines augments TGF-beta1-induced epithelial-mesenchymal transition in A549 cells by up-regulating TbetaR-I. Cell motility and the cytoskeleton.

[40] Luo DD, Fielding C, Phillips A, Fraser D (2009). Interleukin-1 beta regulates proximal tubular cell transforming growth factor beta-1 signalling. Nephrology, dialysis, transplantation: official publication of the European Dialysis and Transplant Association - European Renal Association.

[41] Ginestier C, Liu S, Diebel ME, Korkaya H, Luo M, Brown M (2010). CXCR1 blockade selectively targets human breast cancer stem cells in vitro and in xenografts. The Journal of clinical investigation.

[42] Kim SY, Kang JW, Song X, Kim BK, Yoo YD, Kwon YT (2013). Role of the IL-6-JAK1-STAT3-Oct-4 pathway in the conversion of non-stem cancer cells into cancer stem-like cells. Cellular signalling.

[43] Korkaya H, Liu S, Wicha MS (2011). Regulation of cancer stem cells by cytokine networks: attacking cancer's inflammatory roots. Clinical cancer research: an official journal of the American Association for Cancer Research.

